# Rheo-Kinetic Study of Sol-Gel Phase Transition of Chitosan Colloidal Systems

**DOI:** 10.3390/polym10010047

**Published:** 2018-01-05

**Authors:** Piotr Owczarz, Patryk Ziółkowski, Zofia Modrzejewska, Sławomir Kuberski, Marek Dziubiński

**Affiliations:** 1 Department of Chemical Engineering, Lodz University of Technology, Lodz 90-924, Poland; piotr.owczarz@p.lodz.pl (P.O.); marek.dziubinski@p.lodz.pl (M.D.); 2 Department of Environmental Engineering, Lodz University of Technology, Lodz 90-924, Poland; zofia.modrzejewska@p.lodz.pl; 3 Department of Molecular Engineering, Lodz University of Technology, Lodz 90-924, Poland; slawomir.kuberski@p.lodz.pl

**Keywords:** chitosan, hydrogel, phase transition, gelation mechanism

## Abstract

Chitosan colloidal systems, created by dispersing in aqueous solutions of hydrochloric acid, with and without the addition of disodium β-glycerophosphate (β-NaGP), were prepared for the investigation of forming mechanisms of chitosan hydrogels. Three types of chitosan were used in varying molecular weights. The impacts of the charge and shape of the macromolecules on the phase transition process were assessed. The chitosan system without the addition of β-NaGP was characterized by stiff and entangled molecules, in contrast to the chitosan system with the addition of β-NaGP, wherein the molecules adopt a more flexible and disentangled form. Differences in molecules shapes were confirmed using the Zeta potential and thixotropy experiments. The chitosan system without β-NaGP revealed a rapid nature of phase transition—consistent with diffusion-limited aggregation (DLA). The chitosan system with β-NaGP revealed a two-step nature of phase transition, wherein the first step was consistent with reaction-limited aggregation (RLA), while the second step complied with diffusion-limited aggregation (DLA).

## 1. Introduction

Chitosan is a derivative of chitin that constitutes a building component of crustaceans. Obtained in the deacetylation process, chitosan is a linear polysaccharide consisting of acetylglucosamine and glucosamine molecules, in which the ratio defines the degree of acetylation. The interest in chitosan for biomedical applications results from the biocompatible, biodegradable and non-toxic properties of this compound [1,2,3,4,5,6,7,8]. The main aspects of chitosan use in biomedicine are smart drug delivery systems and scaffolds in tissue engineering [9,10,11,12,13]. Both of these uses employ the phenomenon of sol-gel phase transition of chitosan hydrogels.

Due to the mutual association of chitosan molecules, this compound is not water-soluble [14]. Obtaining a soluble system is possible using water solutions of organic and non-organic acids. Solubility in such a system is caused by the protonation phenomenon of free amino groups H^+^ + –NH_2_ ↔ –NH^3+^ [15,16]. Chitosan molecule properties change from hydrophobic to hydrophilic. The association forces are overcome, which enables the hydration of polymer chains. A colloidal system (sol) is formed in which the polymer chains become dispersed in a continuous medium—a water solution of the acid. As a result of the changes in physical or chemical parameters (e.g., pH, temperature, polymer concentration) of the chitosan solution, the system undergoes a phase transition to the form of a hydrogel [17,18,19,20].

Chitosan macromolecules may form various shape configurations in solutions specified with parameter *a* in Mark Houwink’s equation. The shapes of macromolecules assume the form of stiff, impenetrable spheres (*a* < 0.5), flexible balls enabling the flow of the solvent (0.5 < *a* < 1.2) and disentangled, stiff rods (*a* > 1.2). Using this type of solvent and the related differences in the ionic power and pH of the solutions lead to various shape configurations of the polysaccharide macromolecules in the solution [21,22,23,24]. This results from the equilibrium between the intermolecular interactions, mainly electrostatic repulsion, hydrogen bonds and mutual association of the polymer molecules. The molecular weight of chitosan also influences the configurations of the chain structures, as the charge distribution of the functional groups changes with the molecular weight [23].

The necessity to use aqueous acid solutions as a chitosan solvent and the connected acid reaction of the chitosan systems excluded certain biomedical uses. What turned out to be an answer was systems with an addition of substances neutralizing the reaction of the solutions and allowing to maintain a pH close to the physiological value of a human body (pH~7). In the literature, the most frequently used is disodium β-glycerophosphate (β-NaGP). These systems are characterized by a neutral reaction and a thermo-induced gelation mechanism [18,25,26].

When temperature increases, a reduction of positively charged amino groups occurs, which is caused by a decrease of chitosan pK_a_. The released H^+^ protons are captured by the dissociated β-GP^2−^ groups. The charge neutralization of the chitosan molecules results in a vanishing of electrostatic repulsion and polymer molecules change their character from hydrophilic to hydrophobic. Coagulation of polymer molecules begins, induced by hydrophobic effects and hydrogen interactions and an initiation of the gelation process occurs. The addition of β-NaGP functions as a buffer and acceptor for the protons released in the gelation process in the system. It was also stated that β-NaGP does not participate in building a crosslinked structure. This was proven by experiments in which β-NaGP freely diffused from a prepared gel structure [27].

In the literature [28], chitosan gels are considered as physical hydrogels, in which a lattice is created by hydrogen bonds among amino groups. However, there is no explicit answer as to which processes take place, when the samples are heated and a structure is formed in them in the case of acid reaction solutions (chitosan salt solutions created with ions of an acid solvent) and what processes take place in the case when a buffering substance is added to the solution.

The aim of the paper was to specify the mechanism of forming chitosan hydrogels from aqueous solutions of hydrochloric acid with and without the addition of disodium β-glycerophosphate (β-NaGP). Characteristic parameters of the process of phase transition, induced by a temperature increase, were determined.

For this purpose, the changes in storage and loss moduli as a function of temperature were determined using oscillation rheology methods. The influence of shearing on the deformation of polymer chains was tested. The pH values and the Zeta potential of the tested chitosan solutions were specified.

## 2. Materials and Methods

### 2.1. Materials

For the present study, three types of chitosan were used varying in molecular weight: Chitosan from the Fluka company of unknown origin (sample 1), product No. 50949, lot No. 1078112; chitosan from the Sigma-Aldrich^®^ company (Sigma-Aldrich Sp. z o.o., Poznan, Poland) obtained from crab shells (sample 2), product No. 50494, lot No. 0001424218; chitosan from the Sigma-Aldrich^®^ company obtained from shrimp shells (sample 3), product No. 50494, lot No. BCBB5837.

### 2.2. Preparation of Chitosan Solutions

Chitosan solutions (2.5% *w*/*w*) were prepared by dispersing 0.4 g chitosan in 16 g 0.1 M HCl solution. The obtained solutions were characterized by an equimolar number of H^+^ ions and –NH_2_ amino groups in chitosan molecules. After dispersing, the container with the sample was covered (in order to prevent vaporization) and left for 24 h at room temperature. After that time, the sample was left at the temperature of 5 °C for 2 h. The above procedure was repeated twice for each type of chitosan in order to investigate two sets of solutions: Chitosan-solvent and chitosan-solvent-buffer. The second set was prepared by addition of disodium β-glycerophosphate (β-NaGP). First, the buffer in the amount of 2 g was dissolved in 2 g of distilled water and then the resulting solution was gradually added to a cooled solution of chitosan.

### 2.3. Materials Characteristics

The degree of acetylation (DA) was determined by titrimetric method. The pH measurements were made in a heated bath with the use of a pH-meter ELMETRON CP-401 (ELMETRON Sp. j., Zabrze, Poland), equipped with an electrode for viscous liquids ERH-12-6. The molecular weight (M_w_ and M_n_) of the tested samples was determined by gel permeation chromatography (GPC/SEC) with the use of the high-performance liquid chromatography (HPLC) on a KnauerSmartline company (Berlin, Germany) apparatus, equipped with an analytical isocratic Pump1000 and DRI detector (S-2300/2400, Knauer). The Zeta potential was measured on the basis of electrophoretic mobility of the molecules in an electric field by means of Malvern ZetaSizer Nano ZS (Malvern Instruments Ltd., Malvern, UK). The measurements were conducted at a steady temperature of 30 °C using a folded capillary cell DTS1060 type. The changes in the Zeta potential during the heating of the sample were also established. In order to do so, measurements were made on one sample placed in a cell at the temperature of 20 °C and the value of the Zeta potential was established every 2 °C while heating the sample to 40 °C. The basic properties of chitosan solutions samples are presented in Table 1.

### 2.4. Rheological Measurements

The rheological properties of the chitosan solutions were measured using rotational rheometer Anton Paar Physica series MCR 301 (Anton Paar, Warszawa, Poland). The viscous properties of the tested samples were established in a cone-plate system, of 50 mm diameter, 1° cone angle and 47 µm cone truncation. The flow curves in the range of shear rates 0.01 s^−1^ < γ̇ < 500 s^−1^ were established. A sample, placed at the temperature of 5 °C, was heated at the rate of 1 deg/min to 20 °C. After that, subsequent flow curves were made at steady temperatures of 20, 25 30, 35 and 40 °C using the same heating rate between measurement intervals.

The viscoelastic properties of chitosan systems were established in the same measurement system. The measurement was conducted in the linear range of viscoelasticity at low amplitude of deformations γ = 1% and constant angular frequency ω = 5 rad/s. The gelation process was conducted in non-isothermal conditions, at a constant heating rate of 1 deg/min, from the temperature of 5 °C (storage temperature) to 60 °C. Data of temperature ramp tests are presented as an average from three measurements for each sample. The free surface of the sample was covered with thin layer of low-viscosity silicone oil to prevent sample evaporation during temperature sweep tests [18,20].

The impact of the shear rate on the deformation of a sample was conducted at the temperature of 5 °C and the method of a three-interval thixotropy test was applied (three-interval thixotropy test (3ITT) cillations—rotations—oscillations). In the middle interval, the sample was deformed at a constant shear rate (10^1^, 10^2^ and 10^3^ s^−1^). In the initial and final interval, the sample was tested by means of oscillatory shear at a constant deformation amplitude γ = 1% and angular frequency ω = 5 rad/s in order to establish the properties before and after the rotational deformation process.

## 3. Results

### 3.1. The pH and the Zeta Potential of Chitosan Solutions

**Figure 1:**
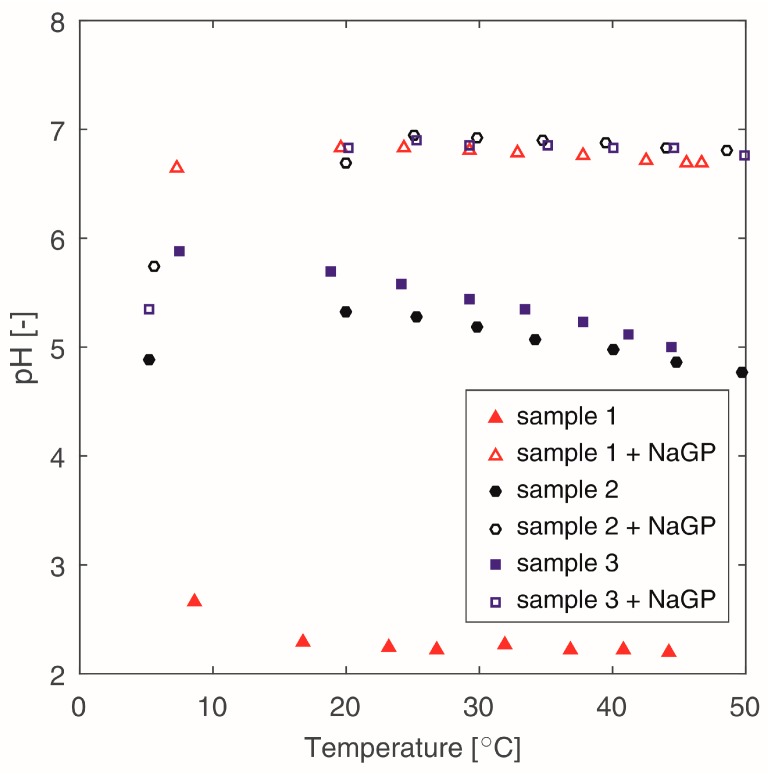
Influence of the temperature on the pH value for different chitosan solutions.

The pH values of chitosan solutions in the temperature function are presented in Figure 1. A significant influence of the addition of β-NaGP on the pH value was observed. Dissociation of this compound in the solution creates the conditions of equilibrium between β-GP^2−^ ions and positively charged –NH^3+^ amino groups, increasing the pH value of the solutions. For all samples containing the buffer, the pH values of the solutions oscillate around a neutral reaction (pH~7) and are independent of the temperature, excluding the pH values for T < 10 °C (Figure 1).

Chitosan solutions without the addition of the buffer show an acidic pH. The lowest pH value occurs for sample 1 and is approximate to the pH of a pure solvent (0.1 M HCl). Correlations between the molecular weight of chitosan and the pH of the solutions for systems without the addition of β-NaGP can be observed. For lower molecular weight of chitosan, the pH values of the solution are lower. An acid reaction of the solution suggests that a major part of the –NH^2^ amino group was not protonated, despite an equimolar number of these groups and H^+^ ions. This can be justified with the configuration taken by chitosan macromolecules in a solution. In a case when a macromolecule has the form of a stiff, entangled ball, the access to the amino groups is difficult. Protonation occurs only outside the ball. In a case when the configuration of a macromolecule has a more disentangled (flexible) form, protonation can take place inside the macromolecule.

The presence of the above-mentioned configurations in the tested solutions is confirmed by the values of the Zeta potential presented in Table 1. For the samples without the addition of β-NaGP, the Zeta potential assumes values above 30 mV (the verge of colloidal stability). Thus, in these solutions the chitosan macromolecules have the form of a ball suspended in a dispersion medium. The hydrophobic core and the positively charged external layer of the macromolecule enable the solvation by water molecules. On the other hand, values of the Zeta potential for solutions with an addition of β-NaGP are ca. 10 times lower and are far below the verge of stability. This probably results from the more flexible and disentangled form of the macromolecule, allowing solvent penetration into the core and as a consequence this results in distribution of the charge not only on the external layer but also inside the macromolecule.

**Figure 2:**
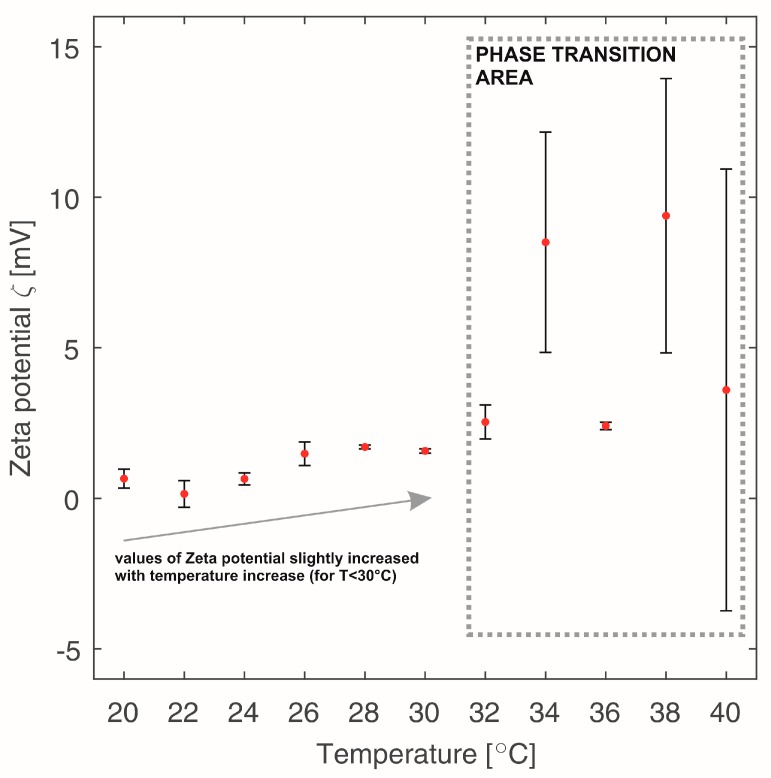
Zeta potential values determined during heating of sample 2 with addition of β-NaGP. Error bars represent the standard deviation.

Analysis of the changes in the Zeta potential values obtained during the heating of the samples containing β-NaGP (Figure 2) shows that during the test, the samples showed unstable properties in a transitional form between a sol and a gel. At the temperature below 30 °C, the measured values of the Zeta potential gradually increase. With the time increasing, a decrease of ionic strength of the amino groups takes place and thus in the polymer chains properties change from hydrophilic to hydrophobic, combined with a disentangling of the chitosan macromolecules. After exceeding the temperature of 30 °C, the sol-gel equilibrium moved towards the creation of a polymer network—a gel structure. Due to this, any further measurements of the Zeta potential are encumbered with errors. This is a consequence of the measuring technique of the equipment—the measurement of the electrophoretic mobility of the molecules. Due to a limited mobility of the molecules trapped in the lattice structure, erroneous readings of the measured values occur. It is visible in Figure 2 as a cloud of scattered spots—high standard deviation. This also explains the high values of measurement errors of the Zeta potential values for all samples containing β-NaGP, which is presented in Table 1.

### 3.2. Influence of Temperature on the Viscous Properties of the Chitosan Solutions

**Figure 3:**
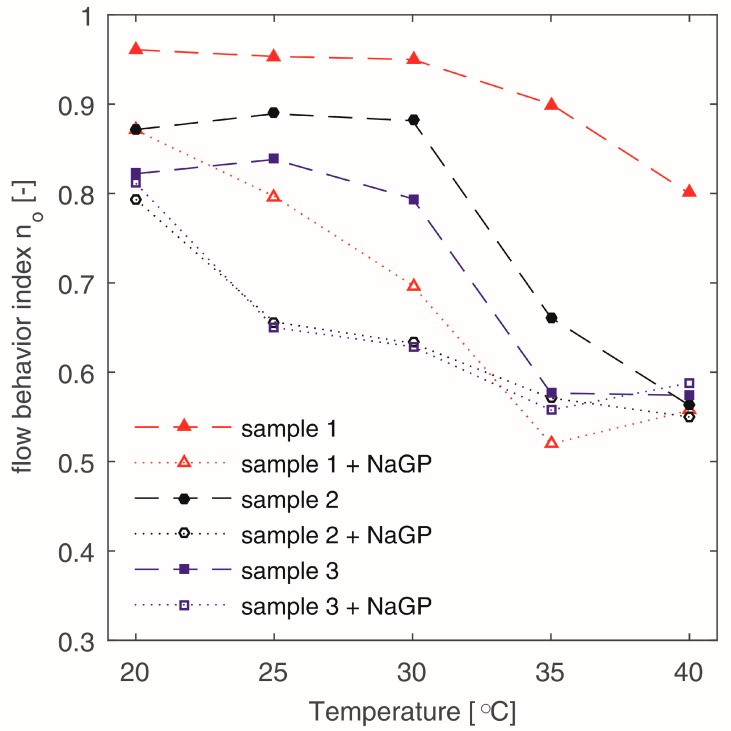
Influence of the temperature on the flow behaviour index n_o_ values for different chitosan solutions (the lines are only for visual aid).

The flow curves of the chitosan solutions allow us to state that in the investigated temperature range, all the tested samples show properties of non-Newtonian shear thinning fluid. In Figure 3, changes in the values of flow behaviour index n_o_ of Ostwald-de Waele power law model were presented for samples containing β-NaGP and without the addition of a buffering substance. For all the tested samples, an increase of temperature decreases the viscosity of the solvent. Furthermore, an increase of non-Newtonian shear thinning properties can be observed—the value of the n_o_ coefficient decreases. However, the character of those changes depends on the molecular weight of the polymer and the presence of a buffer.

In the case of samples without β-NaGP, the values of the flow behaviour index n_o_ clearly depends on the molecular weight of the polymer; the lower the M_w_ value, the higher the n_o_ value. It could also be observed that the polymer chains assume the configurations of entangled balls. It is confirmed by the Newtonian character of some tested solutions, which is supported by the n_o_ values that are slightly lower than one (Figure 3, sample 1). An increase in the molecular weight and consequently larger balls of more flexible structure [23,29], cause a significant decrease of the value of the n_o_ parameter. During the heating of these samples it was observed that the values of the n_o_ coefficient reveal rapid changes after exceeding the temperature of 30 °C. This is clearly visible for the slopes of the curves assigned to samples 2 and 3 (Figure 3).

The curves characterizing changes in the flow behaviour index of the samples containing β-NaGP have a different slope. These samples show significantly lower values of the n_o_ parameter, already in the temperature of 20 °C. It must be assumed that the addition of the buffer (pH increase, Figure 1) intensifies the disentangling of the polysaccharide chains [22,23] which during shearing arrange more easily in parallel to the shear flow direction—thus, causing an increase in the non-Newtonian properties of the tested liquid.

### 3.3. Influence of Temperature on the Viscoelastic Properties of the Chitosan Solutions

**Figure 4:**
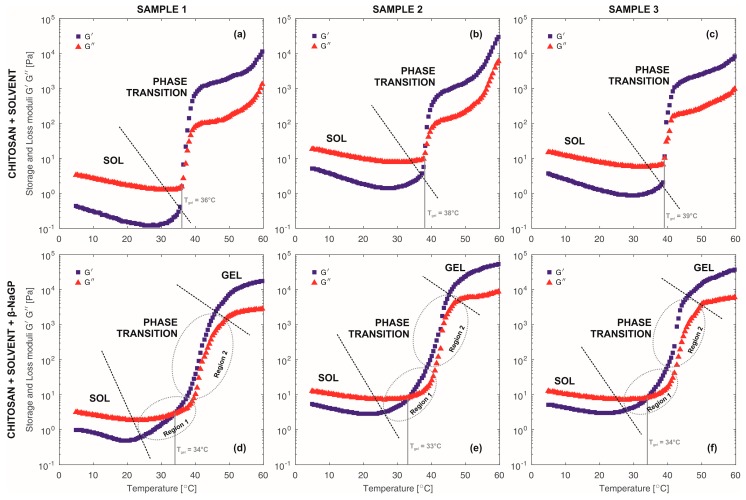
The storage modulus G′ and loss modulus G″ as a function of temperature. (**a**–**c**) Samples without addition of β-NaGP. (**d**–**f**) Samples with addition of β-NaGP.

The evolution of storage modulus G′ and loss modulus G″ as functions of temperature is shown in Figure 4, for systems with and without the addition of β-NaGP. At the initial stage, both systems reveal sol-like behaviour, showing a typical behaviour for a viscoelastic liquid. The loss modulus dominates over the storage modulus. At the same time the values of both moduli and thus the complex viscosity decreases with the increase of temperature and prove the general dependency of liquid viscosity decrease with the temperature increase. In accordance with the Stokes-Einstein equation [30], with a temperature increase the diffusion coefficient increases and an intensification of Brownian motion takes place. Chitosan solutions at this stage are characterised by charged polymer chains. This charge stabilizes the systems and prevents crosslinking [20,31].

With a temperature increase the phase transition occurs—the values of the storage and loss moduli increase. This is a result of the neutralization of the charge of chitosan and formation of a crosslinked structure (gel). Comparing the two types of systems (with and without β-NaGP), a difference in the dynamics of the moduli changes can be noticed. A more rapid increase of moduli for solutions without the addition of β-NaGP compared with the solutions containing the buffer can be observed. Additionally, in the chitosan-solvent-β-NaGP systems, two regions of phase transition can be distinguished in contrast to chitosan-solvent systems where there is one rapid increase of storage modulus.

In the final stage, dominance of storage modulus over loss modulus indicate the gel-like behaviour. However, the plateau values of moduli in chitosan-solvent systems are one decade lower compared to second systems. Furthermore, this plateau is not stable after exceeding a temperature of 50 °C in contrast to chitosan-solvent-β-NaGP systems. The other difference between two systems is revealed as a slightly higher phase transition temperature for chitosan-solvent systems.

In the initial phase of the temperature ramp experiments, the stability of the solutions is conditioned by the hydrophilic character of the chitosan molecules (Chit) related with the presence of positively charged –NH^3+^ groups. The protonation reactions of the amino groups in both solution types are presented below:

Chit^0^ + H^+^ + Cl^−^ → ChitH^+^ + Cl^−^

2Chit^0^ + 2H^+^+2Cl^−^ + 2Na^+^ + GP^2−^ → 2ChitH^+^ + 2Na^+^ + 2Cl^−^ + GP^2−^

Analysing the systems of chitosan-β-NaGP [25,26], a decrease in chitosan pK_a_ was proved with an increase in temperature and at the same time an impact of temperature on pK_a_ of β-glycerophosphate was not observed. Accordingly, an increase in temperature (supplying energy) causes a release of protons and a reduction in the charge on the chitosan molecule. The released H^+^ protons are connected to a negatively charged GP^2−^ ion [26]. Repulsive electrostatic interactions among the chitosan molecules vanish. Chitosan interchain attractive interactions resulting from hydrophobic effects and hydrogen interactions begin to dominate. The reactions taking place during phase transition are presented below:

ChitH^+^ + 2Na^+^ + Cl^−^ + GP^2−^ → Chit^0^ + 2NaCl↓ + H_2_GP

The basic factor leading to the gelation of chitosan systems is a reduction of the charge on the polymer molecule, induced by supplying energy. The presence of β-NaGP in the role of an acceptor of the protons released from the chitosan causes lowering of the phase transition temperature. In acid systems, with no buffer present, phase transition is heavily dependent on the diffusion of the polymer molecules. For this reason, the phase transition process requires a greater amount of supplied energy (a higher temperature).

### 3.4. Non-Isothermal Gelation Kinetics

An analysis of the changes in the values of the storage modulus G′ and the use of kinetics model of polymer crystallization which is based on the Arrhenius equation, allows to determine the activation energy for the gelation process [32,33,34]. An equation describing the kinetics of a gelation process taking place with a temperature increase takes the following form [32]:

(1) $$\ln\left(\frac{1}{{G^{\prime }}^{n_{a}}}\frac{dG^{\prime }}{\mathrm{dt}}\right)=\ln k_{0}+\left(\frac{E_{a}}{\mathrm{RT}}\right)$$

where exponent n_a_ in the Equation (1), described with the dependency n_a_ = r + s, specifies the order of the polymerization reaction, where r represents the dimension of the growing crystals and s the nucleation type. Parameter r assumes values 1, 2 or 3 respectively for one-, two- or three-dimension structures (rod, disk, sphere), whereas s assumes value 0 for predetermined nucleation (nuclei already present), or 1 for sporadic nucleation (nuclei arise and their number increases linearly with time). In the calculations parameter n_a_ = 2 was assumed on the basis of research presented by authors [18,35], regarding similar polymer systems. The value of dG′/dt in the Equation (1) means the so-called structure development rate. By drawing the dependency ln(1/G′^n^_a_ dG′/dt) vs. 1/T from the curve slope, the value of activation energy E_a_ was determined.

**Figure 5:**
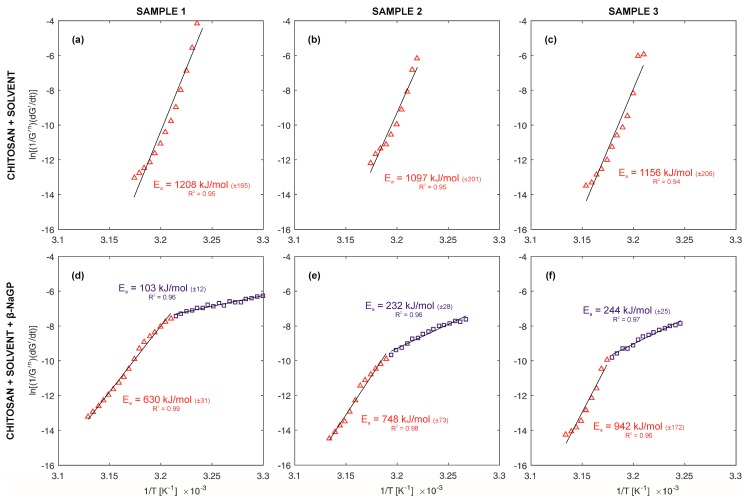
The time-temperature relationship from Equation (1) on Arrhenius plot enabling to determination of activation energy. The straight lines represent the linear approximation of the Equation (1). (**a**–**c**) Samples without addition of β-NaGP. The red triangles represent experimental data from phase transition stage (see Figure 4). (**d**–**f**) Samples with addition of β-NaGP. The blue squares and red triangles represent experimental data from region 1 and region 2, respectively (see Figure 4).

A graphic interpretation of Equation (1) and determined values of activation energies are presented in Figure 5. Comparing the systems with and without an addition of β-NaGP, differences in the amounts of energy inducing the phase transition can be observed. Chitosan-solvent systems required greater energy compared to chitosan-solvent-β-NaGP systems. Furthermore, samples with addition of β-NaGP reveal a two-step nature of phase transition.

### 3.5. Influence of Shear Rate on Polymer Deformations

**Figure 6:**
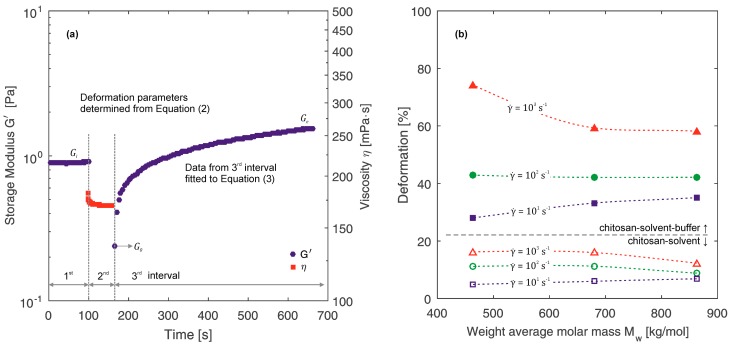
(**a**) Storage modulus and shear viscosity during the three interval thixotropy test for a sample 1 with addition of β-NaGP. (**b**) Deformation parameters as a function of molecular weight for all investigate samples (the lines are only for visual aid).

The impact of shearing deformation on the behaviour of samples in time was specified based on the three-interval thixotropy test (3ITT). The samples were deformed at three different shearing deformations: 10^1^, 10^2^ and 10^3^ s^−1^. On the basis of the obtained measurement results, deformation parameters Def were determined from Equation (2) [36]:

(2) $$\mathrm{Def}=\frac{G_{i}-G_{0}}{G_{i}}$$

where G_i_—value of the storage modulus in the initial stage of the experiment (interval 1), G_0_—value of the storage modulus immediately after the deformation stage (t = 0 s interval 3), see Figure 6a.

Changes in the storage modulus G′ in the recovery stage were fitted to a 2-order kinetic structural model [37,38], assuming G_e_ as an equilibrium value of the storage modulus when t → ∞, k as a constant of the recovery speed and parameter n_a_ = 2 (2-order of the kinetic model):

(3) $${\left[\frac{G^{\prime }-G_{e}}{G_{0}-G_{e}}\right]}^{1-n_{a}}=\left(n_{a}-1\right)\mathrm{kt}+1$$

Figure 6a presents the result of the thixotropy test with a marked characteristic point. The obtained values of deformation parameters for all investigated samples are shown in Figure 6b. Parameters of second order structural model determined by Equation (3) are presented in Table 2. Comparison of the two types of systems indicate that chitosan-solvent-β-NaGP systems reveal stronger thixotropic properties in contrast to chitosan-solvent systems. These differences can result from a shape of molecules. As mentioned above, molecules of chitosan in systems without β-NaGP take the form of stiff, entangled balls. Thus, a shear deformation effect on polymer chains is insignificant. Whereas disentanglement and the more flexible form of chitosan molecules in systems with β-NaGP lead to disentangling of polymer chains due to shear deformation which is revealed by the thixotropic properties.

Furthermore, a correlation between the molecular weight and the degree of deformation can be observed. With a decrease of the value of molecular weight, the degree of deformation of the polymer increases. However, this correlation weakens or vanishes with the decrease of applied shear rate. The effect of molecular weight on the range of deformation parameters values can be also observed. For sample 1 (lowest molecular weight), the difference between maximum and minimum value of deformation parameter is significantly higher compared to sample 3 (highest molecular weight).

## 4. Discussion

**Figure 7:**
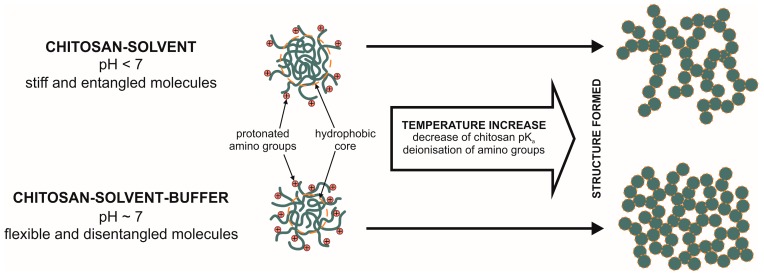
Comparison of the two types of chitosan systems. Shape configuration of chitosan macromolecule and proposed forming of structure.

Analysis of the phase transition process of chitosan hydrogels enables to observe two different mechanisms of this phenomenon. The differences in the composition of the solutions cause the formation of systems varying in pH reaction which influences the shape that the polymer macromolecules take in the solution [22,23,24]. A schematic comparison of the gelation mechanisms occurring in the tested chitosan systems is presented in Figure 7. The figure shows the differences and indicates the characteristic features of the process of structure forming for chitosan-solvent and chitosan-solvent-buffer systems.

Systems of acid reaction (chitosan-solvent) are characterized by macromolecules with a structure of stiff, entangled balls. Protonation of amino groups takes place largely on the external layer of the macromolecules. The determined values of the flow behaviour index n_o_ and the Zeta potential suggest the presence of these macromolecules in the form of dispersed balls surrounded by solvent molecules and the shorter the polymer chain (lower molecular weight), the stiffer the structure of the ball [23,29]. The phase transition temperatures determined in oscillatory tests are included in the range of 36–39 °C. The dynamics of these transitions is a rapid increase in the value of modulus G′ after reaching a certain critical temperature (Figure 4a–c).

Systems of neutral pH reaction (chitosan-solvent-buffer) are characterized by a flexible structure of balls with a possibility of the solvent penetrating inside. The charge distribution, resulting from the protonation of amino groups, takes place both on the external layer and inside the ball. This is indicated by the determined values of the Zeta potential, proving the lack of a double layer. The phase transition temperatures determined in oscillatory and Zeta potential tests are included in the range 33–34 °C. After reaching the critical temperature the value of modulus G′ increases much more slowly than in the case of solutions without β-NaGP (Figure 4d–f). The activation energy of the gelation process indicates higher values for systems of acid reaction in comparison with systems of neutral reaction (Figure 5).

The theory of aggregation processes distinguishes two mechanisms of the phenomenon: diffusion limited aggregation (DLA) and reaction limited aggregation (RLA) [39]. At the initial stage of gelation phenomena, both investigated systems are present in the sol form. Charged chitosan molecules cause a repulsive force between particles [20]. Therefore, the aggregation is limited by repulsive barrier and systems reveal RLA mechanism. As the temperature increases, the motion of particles intensified due to thermal activation. This enables overcoming repulsive barrier. The simultaneous heating of systems reduces the charge of chitosan molecules what follows from neutralization of –NH^3+^ groups [26]. When a certain critical point is reached, the phase transition occurs. Repulsive barrier is overcome by the attractive forces between chitosan molecules.

Comparison of the phase transition stages of both systems indicates differences between gelation mechanism. The chitosan-solvent systems show a rapid increase of storage modulus in a short period of time (Figure 4a–c). It can be assumed that this rapid increase of dynamic moduli results from a vanishing of the repulsive barrier after reaching a critical point. Weakness of repulsive forces lead to an aggregation limited by diffusion (DLA). The chitosan-solvent-buffer systems show a two-step phase transition. The dynamic moduli increase is smoother and occurs in a longer period of time compared with chitosan-solvent system. It can be assumed that in the first period (Region 1 from Figure 4d–f) of phase transition there is a still repulsive barrier between chitosan particles. Thus, the aggregation mechanism is defined by reaction limited aggregation (RLA). However, as time proceeded the repulsive barrier becomes negligible and the aggregation is limited by diffusion (DLA), which can be observed as a rapid increase of dynamic moduli (Region 2 from Figure 4d–f).

## 5. Conclusions

The conducted research revealed that there is a significant impact of the molecular weight on the crosslinked structure of chitosan in an acid solution. The solubility of chitosan depends on amount of H^+^ ions bonding to amino groups [15,16]. In the case of polymers with short chains—low molecular weight—the protonation process inside molecule is inhibited due to the chitosan chains entangling into a stiff ball. Penetration of ions inside the ball is inhibited (sample 1 without β-NaGP). The longer the polymer chain (the higher the molecular weight), the looser is its entanglement. This is proved by both of the results of the conducted tests of the Zeta potential—indicating a decrease of the Zeta potential with an increase of molecular weight (Table 1) and the results of the rheological research—indicating a decrease of the flow behaviour index (transition from Newtonian to non-Newtonian properties) with an increase of molecular weight (Figure 2).

In the case of systems without β-NaGP, a solvation shell is formed around the polymer macromolecules which influences the increase of stability of the system (high values of the Zeta potential). Such a system is hydrophilic and initiation of the aggregation process is significantly inhibited by the solvation shell around macromolecules and existence of repulsive barrier. After exceeding the energy barrier, the phase transition occurs rapidly, which can be explained by the mechanism of diffusion limited aggregation (DLA).

In the case of systems with β-NaGP, two-step phase transition is observed in which the first step is reaction limited aggregation and the second step is diffusion limited aggregation. Occurrence of a first step of the phase transition is a result of buffer addition. The presence of the –GP^2−^ ions, which interact with chitosan amino groups, probably contributes to the formation of the barrier. This barrier needs to be first overcome to enable aggregation between chitosan molecules. After overcoming this barrier, the mechanism of gelation changes from RLA to DLA.

## Figures and Tables

**Table 1:** Properties of chitosan samples.

Chitosan Samples	Weight Average Molar Mass M_w_ (g/mol)	Number Average Molar Mass M_n_ (g/mol)	Polydispersity Index PDI (–)	Degree of Acetylation DA (%)	Zeta Potential ζ (mV) ^1^	
					No Addition of β-NaGP	Addition of β-NaGP
Sample 1	463,000	79,000	5.9	16.8	48.5 (±1.9)	4.74 (±5.3)
Sample 2	680,000	110,000	6.1	18.2	42.3 (±4.4)	1.76 (±4.2)
Sample 3	862,000	145,000	5.9	16.6	37.4 (±2.4)	3.23 (±4.5)

^1^ Zeta potential values expressed as an average of 30 repeated measurements on the same sample at constant temperature 30 °C. Some of a Zeta potential values for samples with β-NaGP were below zero, hence standard deviation values are higher than average values.

**Table 2:** Parameters of second order structural model for all investigated samples.

Chitosan Samples	Shear Rate (s^−1^)	G_0_ (Pa)		G_e_ (Pa)		k (×10^2^)		G_e_/G_0_		R^2^	
		−β *	+β *	−β	+β	−β	+β	−β	+β	−β	+β
Sample 1	10^1^	- **	0.89	-	2.36	-	0.12	-	2.64	-	0.96
	10^2^	0.31	0.75	0.36	1.96	1.23	0.38	1.13	2.64	0.79	0.98
	10^3^	0.28	0.46	0.35	2.13	5.99	0.34	1.31	4.68	0.91	0.99
Sample 2	10^1^	-	5.09	-	7.57	-	0.67	-	1.49	-	0.90
	10^2^	4.06	4.25	4.61	8.18	4.08	1.09	1.13	1.92	0.96	0.96
	10^3^	3.94	3.41	4.73	8.16	5.49	0.96	1.20	2.40	0.94	0.97
Sample 3	10^1^	-	4.93	-	8.87	-	0.68	-	1.80	-	0.93
	10^2^	3.31	3.80	3.76	7.71	1.76	1.35	1.14	2.03	0.96	0.96
	10^3^	3.35	3.27	3.93	7.80	3.21	1.26	1.17	2.38	0.95	0.97

* Samples without addition of β-NaGP (−β), samples with addition of β-NaGP (+β). ** Not possible to fitted data because of the weak phenomenon of deformation.
